# Transformers bridge vision and language to estimate and understand scene meaning

**DOI:** 10.21203/rs.3.rs-2968381/v1

**Published:** 2023-05-29

**Authors:** Taylor R. Hayes, John M. Henderson

**Affiliations:** 1 Center for Mind and Brain, University of California, Davis; 2 Department of Psychology, University of California, Davis

**Keywords:** scene perception, transformer, deep learning, semantics

## Abstract

Humans rapidly process and understand real-world scenes with ease. Our stored semantic knowledge gained from experience is thought to be central to this ability by organizing perceptual information into meaningful units to efficiently guide our attention in scenes. However, the role stored semantic representations play in scene guidance remains difficult to study and poorly understood. Here, we apply a state-of-the-art multimodal transformer trained on billions of image-text pairs to help advance our understanding of the role semantic representations play in scene understanding. We demonstrate across multiple studies that this transformer-based approach can be used to automatically estimate local scene meaning in indoor and outdoor scenes, predict where people look in these scenes, detect changes in local semantic content, and provide a human-interpretable account of why one scene region is more meaningful than another. Taken together, these findings highlight how multimodal transformers can advance our understanding of the role scene semantics play in scene understanding by serving as a representational framework that bridges vision and language.

Semantic knowledge is central to how we perceive and make sense of the complex visual world around us (Biederman, 1972; Potter, 1975; Henderson & Hollingworth, 1999; Wolfe & Horowitz, 2017). While semantic representations are commonly thought of in linguistic terms as the mapping of a word or phrase to a specific object or concept, semantic representations are also thought to help organize perceptual information into meaningful units to efficiently guide our attention in scenes (Henderson, 2007, 2011). Therefore, improving our understanding of the interplay between semantic representations and attention in scenes has the potential to have both broad theoretical impact and to advance a variety of nascent technologies which require rapid scene understanding (e.g., autonomous cars and other agents). While scene semantics are difficult to study and remain poorly understood, recent advancements have made their study more tractable (Henderson & Hayes, 2017; Hayes & Henderson, 2021). Here we take another step toward understanding semantic guidance in scenes by applying a state-of-the-art transformer (Yu et al., 2022) that learns a multimodal vision-language representational space to estimate local scene meaning.

Cognitive guidance theory is the theoretical framework anchoring our work (Henderson, 2003, 2011). Under this view, semantic knowledge stored in memory ‘pushes’ our attention toward scene regions that are recognizable, informative, and relevant to our current goals (Henderson & Hollingworth, 1999; Potter, 1975; Biederman, 1972; Wolfe & Horowitz, 2017; Land & Hayhoe, 2001). That is, where we look in scenes is primarily driven by semantic representations that guide our attention toward meaningful scene regions. There is a long history of evidence supporting the relationship between semantic properties and attention in scenes (Buswell, 1935; Yarbus, 1967; Mackworth & Morandi, 1967; Antes, 1974; Torralba, Oliva, Castelhano, & Henderson, 2006; Williams & Castelhano, 2019), including demonstrations that scene semantics often supplant non-semantic visually salient scene regions (Võ, Boettcher, & Draschkow, 2019; Williams & Castelhano, 2019; Wu, Wick, & Pomplun, 2014). However, one major limitation of much of this earlier work is that it often focused on isolated object-scene semantic relationships (e.g., swapping an octopus and a tractor in an underwater and farm scene respectively). While these discrete semantic manipulations were important in establishing a causal relationship between scene semantics and attention, they do not tell us much about the overall role of semantic guidance in scene understanding (Henderson & Hayes, 2017).

To study the effects of scene semantics globally across entire scenes we recently introduced two different approaches: meaning maps (Henderson & Hayes, 2017) and concept maps (Hayes & Henderson, 2021). Meaning maps use human raters to estimate a given semantic feature at each location in the scene. Specifically, each scene (Fig.1a) is broken into small circular image patches at two spatial scales (Fig.1b), and then participants rate a random subset of these image patches based on a given semantic instruction (e.g., meaningful, informative and recognizable, Henderson & Hayes, 2017). These ratings are then combined back into their respective position to form a map of local scene meaning (Fig.1c). Local scene meaning has repeatedly been shown to be one of the strongest predictors of where people look in scenes regardless of the viewing task (for review see Henderson, Hayes, Peacock, & Rehrig, 2019). In addition to local meaning maps, we also developed a separate language-based approach using a vector space semantic model called ConceptNet Numberbatch (Hayes & Henderson, 2021). ConceptNet Numberbatch derives the semantic relationships between words based on regularities in almost a trillion words of written text and crowd-sourced basic knowledge about the world (Günther, Rinaldi, & Marelli, 2019). The semantic representations from ConceptNet can then be mapped back onto the objects in a scene to form a ‘concept map’ that reflects how semantically related each object is to the rest of the scene, which was also strongly associated with scene attention (Hayes & Henderson, 2021).

Therefore, meaning maps and concept maps each approach scene semantics from a different angle. Meaning maps are constructed by filtering a visual stimulus through the cognitive system of human raters to estimate semantic properties in scenes (e.g., local meaning, Henderson & Hayes, 2017; graspability, Rehrig, Peacock, Hayes, Henderson, & Ferreira, 2020), while concept maps are non-visual, building semantic representations based entirely on regularities in human-generated language. However, humans often acquire semantic knowledge through an interplay of visual and language experience (Clarke, 2015; Ralph, Jefferies, Patterson, & Rodgers, 2017), so scene semantics may best be understood within a computational framework that forms a multimodal mapping between vision and language.

Here we apply just such a framework, a state-of-the-art Contrastive Captioner (CoCa) which serves as a foundational vision-language representational model (Yu et al., 2022). While transformers have played a large role in natural language processing, it is only recently that transformers have been generalized to also include visual and multimodal vision-language domains (Vaswani et al., 2017; Dosovitskiy et al., 2021; Yu et al., 2022). CoCa in particular recently introduced a unique architecture that unifies many of the strengths of previous transformer architectures (i.e., single-encoder, dual-encoder, and encoder-decoder), allowing CoCa to learn aligned unimodal text and image embeddings as well as a fused multimodal image-text representational space (Yu et al., 2022). It is this unique ability that allows CoCa to learn very general representations and achieve state-of-the-art performance across virtually every major image, language, and multimodal benchmark (Yu et al., 2022), and it is precisely this ability that we will leverage to estimate local scene meaning here.

In the present study, we used the pretrained feature space of CoCa to estimate local scene meaning (Fig.1c and Fig.1d) in a model we call ‘DeepMeaning’. The overview of how DeepMeaning estimates local scene meaning is shown in Fig.2a, and can be broadly split into a *feature extraction stage* and a *leave-one-scene-out cross-validation stage*. In the feature extraction stage, we take the CoCa model pretrained on more than 2 billion unique image-text pairs (Fig.2a, purple) and use it to generate CoCa features for each local scene region by breaking each scene into smaller patches using a square grid (Fig.2a, white). Then, we train a linear model (Fig.2a, red) for indoor scenes and a linear model for outdoor scenes where we use these general CoCa features for the scene patches as predictors to estimate local meaning using a leave-one-scene-out procedure (Fig.2a, grey). Indoor and outdoor scenes were modeled separately because there is evidence indoor and outdoor scenes are behaviorally (Torralba et al., 2006) and neurally distinct (Henderson, Larson, & Zhu, 2007). Using this general procedure, we evaluated DeepMeaning based on four criteria: meaning recovery, attention prediction, ability to detect changes in semantic content, and model interpretability (i.e., can we decode in human-interpretable terms why DeepMeaning predicts some regions as higher meaning than others).

We first tested how well DeepMeaning could recover local scene meaning compared to human raters (Fig.2). Using a leave-one-scene-out cross-validation procedure (Fig.2a), DeepMeaning showed excellent recovery at both the individual patch-level (indoor *R*_*cv*_=0.87, Fig.2b and outdoor *R*_*cv*_=0.85, Fig.2c) and for scene-level maps (indoor *R*_*cv*_=0.86, 95%CI [0.84, 0.87]; outdoor *R*_*cv*_=0.76, 95%CI [0.72, 0.79]; Fig.2d). To place DeepMeaning’s scene-level performance in context relative to human raters, when two different groups of human raters rated 40 scenes (34 indoor, 6 outdoor) the scene-level correlation observed between the two rater groups was *R* = 0.87 (95%CI [0.85, 0.89]), which suggests DeepMeaning is performing within or very close to the noise ceiling of human raters (Hayes & Henderson, 2022). Similar to human raters, indoor scenes were more consistently rated by DeepMeaning than outdoor scenes (*t*_*Welch*_(180.45)=5.97, *p* <0.001, 95%CI [0.07, 0.13]), which is reflective of noisier human meaning ratings in outdoor scenes compared to indoor scenes (Henderson & Hayes, 2017).

Next we evaluated whether DeepMeaning maps were strongly associated with where people looked in each scene like human meaning maps (Henderson & Hayes, 2017). Specifically, we correlated the left-out scene DeepMeaning map with a scene fixation density map that summarized where participants looked in that scene (Fig.3a, indoor mean *R*_*cv*_=0.56, 95%CI [0.53, 0.59] and outdoor mean *R*_*cv*_=0.48, 95%CI [0.42, 0.53]) and directly compared this to the correlation observed between human meaning maps and scene fixation density maps (Fig.3b). Overall, DeepMeaning accounted for attention just as well as human meaning maps for both indoor (*t*(132)=−0.83, *p*=0.41, 95%CI [−0.06, 0.02]) and outdoor scenes (*t*(144)=0.09, *p*=0.93, 95%CI [−0.07, 0.07]). Moreover, there was a strong correlation (*R* = 0.86, Fig.3c) of the scene-by-scene attention correlations for DeepMeaning and human meaning, indicating that DeepMeaning and human meaning maps also predicted attention very similarly for a given scene. Finally, we replicated that DeepMeaning maps are strongly correlated with scene attention using 100 indoor and 100 outdoor scenes from an external eye movement dataset (CAT2000, Borji & Itti, 2015, Fig.3d). Again, we found that DeepMeaning maps were very strongly associated with attention for both indoor (*t*(99)=−9180.52, *p* <.001, 95%CI [0.48, 0.52], *d*=918.05) and outdoor scenes (*t*(99)=−7128.11, *p* <.001, 95%CI [0.40, 0.46], *d*=712.81).

Having established that DeepMeaning successfully estimates local scene meaning and DeepMeaning maps strongly correlate with attention, we then tested whether DeepMeaning could detect the removal of local semantic information. To do this we used an adversarial image in which local scene meaning is removed using a diffeomorphic transformation (Stojanoski & Cusack, 2014; Hayes & Henderson, 2022). The diffeomorphic transformation (Fig.4a, 4b) preserves the basic perceptual properties of the scene region while degrading its semantic content. Previously, we have shown that human meaning maps were capable of passing this tough adversarial test, while 3 state-of-the-art deep saliency models failed (Hayes & Henderson, 2022). Therefore, for DeepMeaning to count as an automated method for estimating local scene meaning, DeepMeaning must also be able to pass this strong semantic validity test. To perform the adversarial diffeomorph test, we compared DeepMeaning’s left-out-scene prediction for both the original scene and diffeomorphed scene for this critical altered region. As can be seen (Fig.4c, 4d), DeepMeaning showed a large decrease in estimated meaning for the diffeomorphed region relative to the original unaltered scene region (*t*(39)=18.24, *p* <.001, 95%CI [0.6, 0.75], *d*=2.66). This is an important result, as it establishes that just like human meaning maps (Hayes & Henderson, 2022), DeepMeaning is sensitive to changes in local semantic content.

Finally, we evaluated whether DeepMeaning can go beyond even human meaning maps by providing greater transparency into what underlies its predictions. Given the Contrastive Captioner (CoCa) multimodal backbone of DeepMeaning, we can decode a local scene region into a text caption, providing human-interpretable insight into the model’s representation of a given scene region. As a simple proof-of-concept of this ability, we decoded CoCa’s representation for both the original and diffeomorphed scene patches into text captions (e.g., Fig.4b; also see the supplement for all 40 scene patch comparisons) to understand why the DeepMeaning rating drops in the diffeomorphed region relative to the original in each scene. In all 40 original scene regions semantic content was extracted (e.g., ‘a shelf with many jars of food on it’) with a caption accuracy of 92.5% (37/40), while producing semantically vacuous output for almost all (37/40) of the diffeomorphed image patches (e.g., ‘a circular image of some sort with different colors’), indicating the model struggled to extract semantic content from the diffeomorphed scene regions. A closer examination of the number of total objects correctly recognized, indicated 82 objects were successfully identified in the original scene regions, while only 5 objects were correctly recognized in the diffeomorphed scene regions captions. This 94% drop in objects extracted provides a clear explanation for the large 2.6 standard deviation drop in the DeepMeaning ratings we observed when a region was diffeomorphed: when the amount of semantic content represented plummets, so does the DeepMeaning rating. This simple demonstration shows the promise offered by a multimodal representational space that provides a human-interpretable bridge across vision and language.

Understanding the role semantic representations play is central to understanding the role that cognitive guidance plays in scene understanding. Previous work has approached this problem by measuring direct human behaviors (i.e., semantic ratings of images and eye movement behavior relative to semantic feature manipulations) or by estimating human semantic representations based on regularities in large text corpora. Both approaches are useful, but they leave a representational gap that makes it difficult to understand the precise mapping between visual input and semantic knowledge, either because they are filtered through the human brain or because they are only based on a single representational space without a mapping to the other. Our work here shows that bridging vision and language representational mappings not only provides an automated way to accurately estimate scene meaning and attention, but perhaps more importantly, a means to interpret the representational embeddings that underlie those predictions. More broadly, the current study serves as another piece of evidence that multimodal transformers like CoCa can serve as ‘foundational’ vision-language models for downstream tasks (Yu et al., 2022).

In summary, we used a state-of-the-art transformer trained on billions of image-text pairs to reveal how joint representations learned from vision and language can predict what scene regions people find meaningful and consequently where they look. We demonstrated that this computational framework successfully recovers human meaning ratings near ceiling, transfers as a strong predictor of scene attention, detects local changes in semantic content, and provides a direct route to human-interpretability via multimodal image-text decoding. The ability to offer automated scene meaning and attention prediction using a joint representational space that bridges vision and language has tremendous potential for advancing our understanding of how semantic representations produce rapid scene understanding with implications for cognitive science, computer vision, linguistics, robotics, and artificial intelligence.

## Methods

### Contrastive Captioner (CoCa)

#### Model implementation.

We used the OpenClip Contrastive Captioner (CoCa) implementation (coca_ViT-L-14 with the *mscoco_finetuned_laion2b_s13b_b90k* pretrained weights, Ilharco et al., 2021) based on the original CoCa model by Yu et al. (2022). The OpenClip CoCa model was pretrained on 13 billion samples from the LAION-2B dataset using a batch size of 90,000, a learning rate of 1e-3, and a cosine decay learning rate schedule (Schuhmann et al., 2022). These weights were then finetuned using the Microsoft COCO dataset (Lin et al., 2014) using a batch size of 128, a learning rate of 1e-5, and a cosine learning rate schedule (Schuhmann et al., 2022).

#### LAION-2B data.

The LAION-2B dataset is the English subset of the larger multilingual LAION-5B dataset. The LAION-2B dataset is an open dataset for model training that contains 2.32 billion image-text pairs (Schuhmann et al., 2022).

### DeepMeaning

#### Architecture.

**Figure 1.**
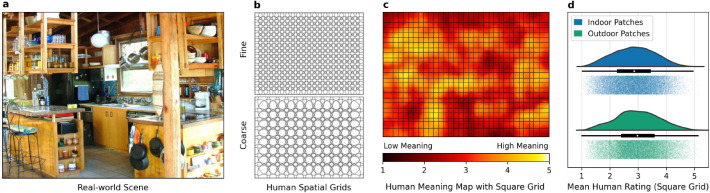
Meaning mapping and input/target preprocessing. A meaning map for each scene (a) is built by breaking each scene into circular patches at two spatial scales (b), and then having humans rate the patches. The human patch ratings are then recombined to generate a scene meaning map (c). To train DeepMeaning, each scene image (a) and meaning map (c) were broken into patches using a square grid (c). The square scene image patches served as the input to the pretrained Vision transformer (ViT) of the Contrastive Captioner (CoCa) while the average meaning map value of each square region served as the target value to be predicted. Raincloud plots of the distribution of the meaning target values for indoor and outdoor scenes were normally distributed (d).

DeepMeaning is composed of two components: a pretrained Contrastive Captioner (CoCa) transformer that is used as a feature extractor and a linear regression model that is trained to use these features to predict scene meaning. Specifically, the pretrained weights learned by the Contrastive Captioner by training on the LAION-2B dataset were frozen, and then used to extract general features from each square scene image patch. The extracted image patch features and their corresponding meaning ratings (Fig.1c and Fig.1d) were then used to train a linear regression model to predict meaning ratings for indoor and outdoor scene patches separately using a leave-one-scene-out cross-validation procedure.

#### Square grid, scene patches, and meaning rating preprocessing.

Each scene and its corresponding meaning map were split into 96×96 pixel square patches with 35% overlap (Fig.1c). Each square scene image served as an input to the vision transformer (Vit) component of CoCa for feature extraction. The meaning value for each square scene region was computed as the average across its location in the corresponding human meaning map and served as the target value to be predicted (Fig.1d).

#### Leave-one-scene-out cross-validation procedure.

A leave-one-scene-out train/test cross-validation procedure was used to estimate the generalization performance of DeepMeaning. In this procedure, the linear regression model component of DeepMeaning was trained on all scenes but one, and then the trained regression model weights were frozen and used to predict the meaning values for the left-out-scene image patches. This procedure was done separately for indoor and outdoor scenes producing a separate set of linear weights for each scene type.

The diffeomorph dataset and CAT2000 dataset were special cases that required a slightly modified cross-validation procedure. For the diffeomorph dataset, in addition to testing on the original scene, the trained linear model was also tested on the diffeomorphed version of the left-out scene. For the CAT2000 dataset, since our meaning training set of 281 scenes did not contain any of the CAT2000 scene images, the indoor and outdoor linear models were trained on all the meaning patch ratings from either the 136 indoor or 145 outdoor scenes respectively.

### Meaning map data

#### Participants.

University of California, Davis undergraduate students (N=1149) with normal or corrected-to-normal vision participated in the meaning rating study in exchange for course credit. All participants were naive concerning the purposes of the experiment and provided verbal or written informed consent as approved by the University of California, Davis Institutional Review Board. All experiments were performed in accordance with relevant guidelines and regulations.

#### Stimuli.

281 real-world scene images were meaning mapped. The 281 scenes consisted of a mix of indoor (136) and outdoor (145) scenes and included scenes from 100 unique scene categories (e.g., kitchen, office, park, street, etc.).

#### Meaning mapping procedure.

**Figure 2.**
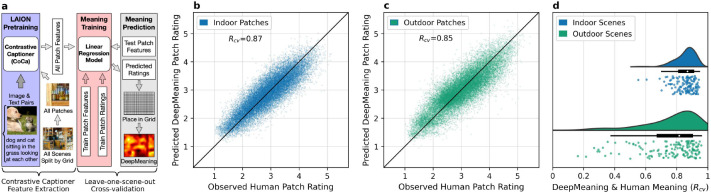
DeepMeaning overview and meaning recovery results. DeepMeaning combines the features from a Contrastive Captioner (CoCa) transformer pretrained on billions of image-text pairs with a linear model to predict patch meaning ratings (a). The scatterplots (b-indoor, c-outdoor) show DeepMeaning’s patch-level meaning prediction relative to human meaning ratings where each dot in the plot represents an individual scene patch. The raincloud plots (d) show the distribution of the correlations between the DeepMeaning predicted meaning map and the ground truth human meaning map, where each dot represents a left-out indoor or outdoor scene.

Meaning maps were generated for each scene using the same meaning mapping procedure and same rating instructions as Henderson and Hayes (2017) (see https://osf.io/654uh/ for the code and complete rating instructions). Specifically, a meaning map was created for each scene by cutting the entire scene into a dense array of overlapping circular patches (Fig.2b) at a fine spatial scale (300 patches, diameter=87 pixels) and coarse spatial scale (108 patches, diameter=205 pixels). Human raters then provided ratings of 300 random fine or coarse scene patches based on how informative or recognizable they thought they were on a 6-point Likert scale (Henderson & Hayes, 2017; Mackworth & Morandi, 1967). Patches were presented in random order and without scene context, so ratings were based on context-independent judgments. Each unique patch was rated by three unique raters.

A meaning map (Fig.1c) was generated for each scene by averaging the patch rating data at each spatial scale separately, averaging the spatial scale maps together, and then smoothing the grand average rating map with a Gaussian filter (i.e., Matlab ‘imgaussfilt’ with *σ* = 10, full width at half maximum=23 pixels).

### Eyetracking data

#### Internal.

Eye tracking data from previous studies (Hayes & Henderson, 2021; Henderson & Hayes, 2017; Henderson, Goold, Choi, & Hayes, 2020) within our lab were used to validate DeepMeaning’s ability to predict scene attention. This global dataset contained 136 indoor scenes and 145 outdoor scenes. Each scene was viewed for between 6 and 12 seconds by between 50–65 observers during a scene memorization task where subjects were told they would later have to perform a scene recognition task. Participant eye movements were recorded using an EyeLink 1000+ tower-mount eye tracker (spatial resolution 0.01°) sampling at 1000 Hz (SR Research, 2010). Participants sat 85 cm away from a 21” monitor and viewed scenes that subtended approximately 27° × 20° of visual angle.

#### External.

One hundred indoor and outdoor scenes from the CAT2000 benchmark eye tracking dataset (Borji & Itti, 2015) served as an external replication of DeepMeaning’s ability to estimate local meaning that transfers to predict scene attention. Each scene in the CAT2000 dataset was freely-viewed by 24 observers for 5 seconds while their eye movements were recorded using an EyeLink 1000 eye tracker(SR Research, 2010).

### Diffeomorph data

The diffeomorph scene set from Hayes and Henderson (2022) was used to assess whether DeepMeaning could successfully detect the local removal of semantic content from a scene. The diffeomorph dataset contained 40 scenes in two conditions: diffeomorphed and original. In the diffeomorph condition, a diffeomorphic transformation (Stojanoski & Cusack, 2014) was applied to one local region in each scene to remove the semantic content from that region while preserving its image features (Hayes & Henderson, 2022). In the original condition, the scenes were presented unaltered. Human meaning ratings were then collected for both the original scenes (N=164) and the diffeomorphed scenes (N=164) using the same *Meaning Mapping Procedure* described above.

Captions for the original and diffeomorphed patches were decoded from CoCa using a top 5% quantile token generation type with a temperature of 1 and a repetition penalty of 2 (Ilharco et al., 2021). Each caption was evaluated based on whether it accurately described the content of the scene patch (yes or no) and how many objects it correctly recognized in each patch.

## Figures and Tables

**Figure 3.**
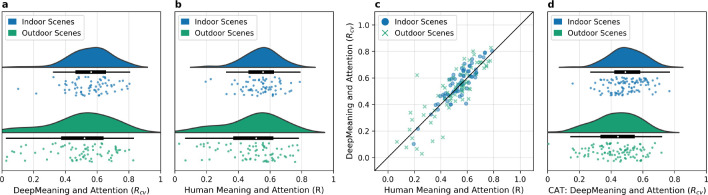
DeepMeaning maps transfer to predict scene attention just like human meaning maps. Raincloud plots show that DeepMeaning maps (a) and human meaning maps (b) both correlate strongly with scene attention. Moreover, the correlations between human meaning maps and attention and DeepMeaning maps and attention were very similar (*R*_*cv*_=0.90) scene to scene (c). Finally, we applied DeepMeaning to an external scene dataset (CAT2000) and replicated a strong correlation between DeepMeaning maps and scene attention (d) for both indoor and outdoor scenes.

**Figure 4.**
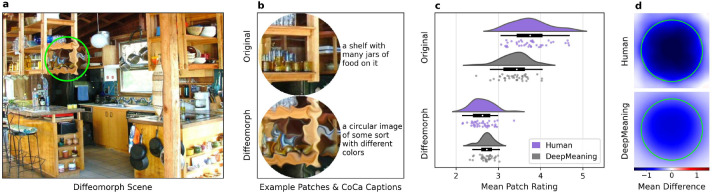
DeepMeaning detects the removal of semantic content. We applied DeepMeaning to adversarial scenes where semantic content was removed while preserving image features via a diffeomorphic transform (a,b). DeepMeaning passed this adversarial test just like human meaning maps, showing a large decrease in meaning value for the diffeomorphed scene region relative to the original non-diffeomorphed scene region (c,d). Additionally, we used CoCa to decode captions for the original and diffeomorphed image patch (b), revealing that CoCa could no longer identify a mapping between semantically meaningful objects, offering a human-interpretable explanation for the drop in meaning values.
